# Supplementary material for: Are carbon emissions trading and green financial instruments synergistic? -Comprehensive quantitative research based on content analysis
Source: PLoS One. 2024 Mar 7;19(3):e0298601. doi: 10.1371/journal.pone.0298601 (PMC10919601; doi:10.1371/journal.pone.0298601)
Supplement: S1 Data — (ZIP) [file pone.0298601.s001.zip › Data/地级市Z_F工作报告.zip!/─■╧─/╓╨╬└╩╨/└┤╫╘╣1⁄2╓┌║┼í╛┬φ┐╦╩2╛▌═°í┐_2007.docx]

各位代表：

现在，我代表市人民政府，向大会作政府工作报告，请予审议，并请政协委员和其他列席人员提出意见。

一、2006年工作回顾

刚刚过去的一年，是实施“十一五”规划的开局之年，也是我市经济社会发展的攀升之年。一年来，市人民政府在自治区党委、政府和市委的正确领导下，以党的十六大和十六届五中、六中全会精神为指导，认真落实科学发展观，团结和带领全市人民，在竞争中抢抓机遇，在困难中开拓进取，全面完成了市一届人大三次会议确定的各项目标任务，谱写了跨越式发展的崭新篇章。预计全市地区生产总值完成74亿元，同比增长12%。其中：第一产业14.8亿元，同比增长4%；第二产业28亿元，同比增长18%；第三产业31.2亿元，同比增长10%。全市地方财政总收入4.8亿元，可比增长13.6%。其中：市本级地方财政总收入3.3亿元，可比增长22.1%。全社会固定资产投资65亿元，同比增长18.7%。全市城镇居民人均可支配收入7800元，同比增长8.4%；农民人均纯收入2720元，同比增长6.4%。

——工业经济增势强劲，综合实力大幅提升。全市工业总产值预计完成66.5亿元，同比增长16.4%；工业增加值20亿元，同比增长16.5%。规模以上企业产值、增加值和利税分别为54亿元、16.3亿元和3.2亿元，同比分别增长20%、19%和15%。产值超亿元的企业达到13家、超5亿元的1家、超10亿元的1家。各工业园区和工业基地建设步伐加快，新入园企业48家，累计达到135家。全市共引进各类项目104个，到位资金19.32亿元。与中国冶金科工集团合作，成功重组美利纸业集团公司，林纸一体化项目到位资金18.2亿元，使美利纸业跨入国家大型企业500强行列；引进山东茌平铝业公司，使秦毅集团重新恢复生产。科豪陶瓷和新亚陶瓷地砖、大河数控珩磨机床、昊丰伟业生铁等项目已经建成；中冶美利纸业30万吨涂布白卡纸、10万吨杨木化机浆、2台5万千瓦热电机组和宁夏红集团2万吨枸杞饮料、美康陶瓷地砖、兴尔泰硫酸钾复合肥、西安新盟单晶硅、贺兰山清真羊肉等一批项目正在加紧建设。

——特色产业不断壮大，农村经济稳步发展。全市农业总产值预计完成28.8亿元，同比增长7.4%；农业增加值14.9亿元，同比增长6.4%。特色产业规模进一步壮大。新增枸杞2.4万亩，累计达到18.4万亩；新增设施蔬菜2.4万亩，累计达到8.1万亩；新增压砂地20万亩，累计达到71.8万亩；新增林果7.7万亩，累计达到23.4万亩；种植马铃薯63万亩；种植优质牧草124万亩，全市生猪、牛、羊饲养量分别达到100.8万头、21.9万头、175.2万只。农业标准化进程不断加快。新培育发展农产品流通合作经济组织43个，累计达到155个，年流通额达到8.4亿元；有11类62个农产品获得无公害认定，“香山硒砂瓜”取得有机产品转换证书，“宣和牌鸡蛋”获“宁夏名牌”和“中国名牌”称号，中宁枸杞、香山硒砂瓜、设施蔬菜通过全国绿色食品原料标准化生产基地验收。农业基础设施得到加强。灌区续建配套与节水改造、引黄补灌、山区小流域治理和治河治沟工程成效显著。新农村建设开局良好，启动建设“塞上农民新居”示范点17个。乡村交通环境明显改善，新修乡村道路427公里。

——城市建设快速推进，综合功能明显增强。全市新建、续建城市项目160个，完成投资15.3亿元。其中：城区119个在建和新开工项目完成投资12.5亿元。通过积极争取、多方运作，沙坡头机场隆重奠基，目前各项前期工作正在有序开展。中卫一中、中卫六小、体育训练馆等一批公共项目已经建成；环城东路、环城西路、行政办公区单位办公楼、新区生态湖、生态公园、集中供热、中卫中学等城市重点项目正在建设；实施了“一苑”（沙漠风情苑）、“两湖”（新区生态湖、黄河湿地公园湖）、“六带”（沙坡头大道、迎宾路、彩虹路、怀远路、平安路、应理街景观带）绿化工程，新增绿化面积247万平方米，市区绿化总面积达到376万平方米，人均绿地24.5 平方米。完成了“四街八巷”改造工程，旧城改造步伐加快。严格住房公积金管理，理顺了管理体制。实施房地产项目29个，竣工面积34.9万平方米，居民居住条件极大改善。加强综合环境整治，城市面貌明显改观，被评为2006年度“中国特色魅力城市200强”。中宁县枸杞花园、东苑南区、检察院等18个新建项目和平安街、宁安南街等13个续建项目进展顺利，完成投资2.8亿元。海原县城供水工程、垃圾无害化处理、交通巷等工程投入使用，南门蔬菜市场、汽车站迁建等工程进展顺利。

——第三产业活力增强，财政金融平稳运行。旅游设施建设步伐加快，投资8177万元，实施旅游项目28个，加快了沙坡头旅游区、石空大佛寺等景区景点建设，开发了农家乐等特色旅游项目，形成了一批特色鲜明的旅游精品。宣传促销力度进一步加大，举办了宁夏中卫香山硒砂瓜节、法国人丝绸之路长跑、全区“乡村游”现场会等节事活动。中卫旅游知名度明显提高，沙坡头又被评为“中国最值得外国人去的50个地方之一”。全市接待游客103.2万人次，实现旅游总收入2.6亿元，同比分别增长19.8%和37.9%。商业、服务业、信息、通信等第三产业稳步发展，预计全社会消费品零售总额达到16.4亿元，同比增长14.8%。进一步加大税收征管力度，财源建设初显成效。调整优化财政支出结构，财政保障能力明显提高。加强银政、银企合作，金融信贷规模不断扩大，服务领域进一步拓展。预计全市金融机构存款余额80.8亿元，同比增长18.1%；贷款余额72.8亿元，同比增长13.1%。

——社会事业全面发展，精神文明得到加强。加快科技进步与技术创新，推动高新技术产业化，企业核心竞争力不断增强。深入实施科技特派员创业行动，科普工作取得新成绩。中卫市城区成为宁夏第一个国家可持续发展实验区。争取和实施教育项目30个，总投资6245万元，办学条件进一步改善。素质教育不断深化，教育教学质量稳步提高。海原县和中宁县喊叫水乡通过“两基”验收，全市实现“两基”目标。文化市场秩序进一步规范，文化事业全面发展。全年广场文艺演出182场次，群众文化丰富多彩。全民健身运动蓬勃开展，“体育下乡、篮球进村”工程深入实施，成功举办了全区第三届沙漠体育运动会。中宁县和城区新型农村合作医疗试点进展顺利，公共卫生服务水平和应急能力进一步提高。投资1050万元，改扩建乡镇卫生院13个、标准化村卫生室187个。传染病和地方病防治工作得到强化，计划免疫、妇幼保健工作不断加强。实施农村“户户通电”工程，全市农村实现了全部通电。实施农村广播影视公共服务体系建设项目，建设了城区香山MUDS转发站，使城区香山乡和海原县兴仁镇3万多名山区群众免费收看到8套电视节目。人口和计划生育工作稳步推进，人口自然增长率为9.74‰。环境与资源保护工作成效显著，取缔了城区灌区内10家粘土砖厂、市区267台小锅炉。认真贯彻《公民道德建设实施纲要》，将社会主义荣辱观教育活动与新农村建设有机结合，文明单位、文明村镇等群众性精神文明创建活动成效显著。老龄和残疾人工作进一步强化。加强了民兵预备役建设，支持了驻卫部队建设，强化了军队转业干部、退役士兵安置和拥军优属工作，“双拥”共建活动深入开展。防震减灾、外事、侨务、民族宗教等方面的工作取得新成绩。

——社会保障逐步加强，人民生活有效改善。就业和再就业工作进一步强化，全市新增就业岗位9619个，有769名下岗失业人员实现了再就业，城镇登记失业率为4.2%。争取资金1349万元，解决了21家国有破产改制企业2776名职工的身份置换问题；争取资金786万元，解决了31家国有破产、困难企业1557名离退休职工的医疗保险问题。社会保障事业稳步发展，养老、失业、工伤和医疗保险动态覆盖面进一步扩大。全年累计发放低保资金2682.5万元、各类救灾救济资金1704万元，帮助灾民建房134户537间。清欠农民工工资1601万元，维护了农民工的合法权益。有组织地转移农村剩余劳动力17.8万人，实现劳务收入7.2亿元。其中：海原县输出10.2万人，创收3亿元。完成了南山台吊庄移民工程，搬迁安置移民1200人。27个整村推进村全部验收合格，脱贫人口达到2.03万人。巩固禁牧封育成果，生态环境明显改善。积极筹措抗旱资金，支援山区抗旱，山区群众生产生活得到极大改善。

——民主法制进程加快，行政能力不断提高。自觉接受市人大及其常委会的法律监督和工作监督、市政协及各民主党派、工商联的民主监督、社会各界的群众监督和新闻媒体的舆论监督。全年办理人大代表议案5件、建议35件、政协提案143件。依法行政和政务公开工作稳步推进，制定行政规范性文件17件。深入开展普法教育，全面完成“四五”普法任务，公民法律意识进一步增强。加强经营性土地使用权出让招拍挂，审计监督和行政监察力度不断加大。落实廉政建设责任制，深入开展治理商业贿赂和机关效能建设活动，政府系统廉政建设取得新成效。预防道路交通事故，强化公众聚集场所安全，开展重点行业和领域的安全生产专项整治，全市安全生产形势平稳。重视和加强信访工作，有效化解了各类矛盾纠纷。强化社会治安综合治理，完善治安防控体系，开展“严打”整治行动，努力消除不安定因素，有效维护了社会稳定，实现了“平安市”的创建目标。

各位代表！

过去的一年，我们遇到的困难较多，取得的成绩较好。我们始终坚持发展第一要务，充分发扬“三苦”和“两情” 精神，积极应对国家宏观调控，全面战胜各种自然灾害的严峻挑战，有效抑制经济运行中的不利因素，实现了物质文明、精神文明、政治文明与和谐社会的全面发展。这是自治区党委、政府和市委正确领导的结果，是市人大、市政协及社会各界监督支持的结果，是全市各族人民共同努力的结果，是驻卫各单位、部队积极帮助的结果。在此，我代表市人民政府，向全市工人、农民、知识分子、干部、解放军指战员、武警官兵、公安民警，表示崇高的敬意！向人大代表、政协委员、各民主党派、工商联、人民团体、离退休老同志，向关心和支持中卫发展的社会各界人士，表示衷心的感谢！

在肯定成绩的同时，我们也清醒地看到，全市经济社会发展中还存在着一些矛盾和问题。主要是：经济总量不大，人均总量不高；经济发展和城市建设中的资金、土地等生产要素供需矛盾还较突出；受干旱、疫情影响，部分群众生产生活较为困难；一些部门和单位作风还不够扎实；发展环境还需优化。对于这些困难和问题，我们将高度重视，认真对待，采取有力措施加以解决。

二、2007年主要工作

各位代表，2007年是深入贯彻落实科学发展观、积极推进社会主义和谐社会建设的重要一年。做好今年的工作，意义重大，影响深远。今年，政府工作总的要求是：以邓小平理论和“三个代表”重要思想为指导，深入贯彻党的十六大、十六届六中全会精神，按照自治区党委、政府和市委的工作部署，坚持加快发展第一要务，围绕市第二次党代会确定的奋斗目标，着力推进产业结构调整和转变经济增长方式，着力做强工业、做优农业、做美城市、做活旅游，着力加快山川共济进程，着力推进机关效能建设，切实加强政治建设、文化建设和和谐社会建设，为打造具有实力、富有魅力、文明和谐的新中卫而努力奋斗。主要预期目标是：全市地区生产总值达到83亿元，增长12%。其中：第一产业15.7亿元，增长4%；第二产业33.2亿元，增长18%；第三产业34.1亿元，增长10%。全市地方财政总收入达到5亿元，可比增长13%。其中：市本级地方财政总收入2.8亿元，可比增长13.4%。全社会固定资产投资达到78亿元，增长20%。城镇居民人均可支配收入达到8400元，增长7.7%。农民人均纯收入达到2900元，增长7.4%。

围绕上述目标，重点抓好以下八个方面的工作：

（一）做大做强集群工业，强化构建和谐中卫的经济支撑

把抓工业作为强市的第一战略，坚持“只有大规模、大循环，才能大发展、赚大钱”，创优工业发展平台，壮大特色产业集群，增强自主创新能力，大力发展循环经济，夯实强市富民之基。

培育壮大产业集群。围绕打造全国制浆造纸基地，西北酿酒、陶瓷、机械制造、农副产品加工基地和宁夏冶金化工、建筑建材基地，依托资源优势，加强资源整合，发展壮大造纸、酿酒、冶金化工、建筑建材、农副产品加工、机械制造六大特色产业，打造优势产业集群。以实施造纸、电石、PVC、钢铁“四个百万吨工程”为重点，引导上下游中小企业向“专、精、特、新”方向发展，增强协作配套能力。全年开工建设工业项目70个以上。重点抓好中冶美利纸业20万吨牛卡纸和20万吨高强瓦楞纸、宁夏红集团2万吨枸杞饮料、美康陶瓷1200万平方米地砖、西安新盟年产2000吨单晶硅、赢嘉公司20万吨玉米淀粉等项目建设。着力培植中冶美利纸业、“宁夏红”集团、秦毅集团、大河机床、美康陶瓷、科豪陶瓷、昊丰伟业、通达果汁、赛马中宁水泥等一批竞争实力强、经济效益好的优势企业群体，力争建成产值超20亿元的企业1家、超10亿元的1家、超5亿元的3家、超亿元的10家。全市工业增加值增长18 %以上。

集约发展园区经济。进一步完善美利、宁新工业园区和宁夏红科技园、镇罗金鑫园、常乐陶瓷园等工业基地水、电、路、通信、绿化等配套设施，加速提升承载重大产业化项目的能力，推动园区建设特色化，产业发展集约化。引导新上项目向园区集中，切实提高工业园区和工业基地的投资强度和产出效益，打造以工业园区为中心、以骨干企业为依托、以中小企业为基础的三大工业经济增长极。

增强企业自主创新。鼓励企业制定中、长期发展规划，加强技术改造、技术创新和新产品研发，切实增强自主创新能力。大力发展技术创新中介服务，加速技术引进和科研成果转化。鼓励企业培育有自主知识产权的拳头产品，争创企业品牌和驰名商标。加强工业经济预警预测，协调做好电力、运输等保障工作，确保工业经济平稳高效运行。

积极推进循环经济。把节能降耗摆到更加突出的位置，依法淘汰落后工艺技术和生产能力，改造提升传统产业，努力提高资源综合利用水平、节能降耗水平、环境保护水平，积极发展低消耗、低污染、高利用的循环经济，切实转变增长方式，做到高效、清洁、安全生产，加快构建节约型产业结构，推动工业经济健康快速发展。万元GDP综合能耗降低4%左右。

（二）统筹解决“三农”问题，加快构建和谐中卫的整体进程

以社会主义新农村建设统揽整个农村工作，认真贯彻工业反哺农业、城市支持农村和多予少取放活的方针，严格落实各项支农惠农政策，促进农业不断增效、农村加快发展、农民持续增收。

大力发展现代农业。稳定提高粮食综合生产能力，粮食播种面积稳定在196万亩，总产量保持在42万吨左右。以结构调整为重点培育壮大农业优势特色产业，新增枸杞2.1万亩，累计达到21万亩；新增设施蔬菜2万亩，累计达到10万亩；新增压砂地20万亩，累计达到91万亩；马铃薯种植面积达到80万亩；饲草种植面积稳定在120万亩左右；启动建设宣和东台沙漠生态养鸡示范园区，年内恢复发展100万只，鸡饲养量达到300万只。全市生猪、牛、羊饲养量分别达到125万头、24万头、185万只。做好动物疫病防治，提高抵御各类灾害的能力。健全农产品质量安全体系，加快农业标准化建设，打响绿色品牌，提高市场竞争力。大力培育农产品加工龙头企业，延伸产业链条，增强辐射带动能力，新发展规模以上农产品加工龙头企业10家，累计达到160家。鼓励兴办各类农村专业合作组织，提高农民进入市场的组织化程度，新组建各类流通组织和专业协会25家，总量达到180家。

全力加快山区发展。从解决关系人民群众切身利益的现实问题入手，统筹区域发展，优化资源配置，在山区教育、医疗卫生、劳务输出、特色产业、项目建设等方面与川区进行有效对接，推进以川济山、山川共济。实施避灾抗旱战略，发展山区特色经济，促进山区农民增收。加快发展劳务经济，力争全年劳务输出18万人，实现劳务收入8亿元。加大扶贫开发力度，做好新一轮38个村整村推进扶贫工作，抓好“十万贫困户养羊工程”的滚动发展，年内实现脱贫2.2万人。

着力改善农村面貌。坚持规划先行，完成138个行政村的建设和发展规划。广泛开展以“一池三改”为主要内容的农村环境整治，加快实施“塞上农民新居”、农村危窑危房改造、乡村洁净等工程，硬化乡村道路，以沼气工程带动农村改圈、改厕、改厨，着力推进社会主义新农村建设。新修改造一批通乡通村油路和乡镇客运站，提高乡村道路的通达能力和等级。巩固农村广播影视公共服务体系成果，加快实施广播电视“村村通”工程，使山区34个20户以上自然村群众免费收听到4套广播、收看到8套电视节目。配套完善教育、文体、卫生等设施，不断改善农村生产生活条件。加大农村基础设施投入，加强灌区灌溉管理、农村安全饮水、小流域综合治理工作，加快实施宁夏中部干旱带兴仁农村饮水安全重点供水工程、南山台电灌站改扩建等项目。巩固退耕还林、退牧还草、封育禁牧成果，改善山川生态环境。

（三）加快推进城市建设，提升构建和谐中卫的承载功能

以创建国家卫生城市、国家园林城市、中国优秀旅游城市“三城联创”为抓手，主动融入沿黄城市带发展规划，依托黄河做好水域文章，力争把中卫打造成“河湖泉一水贯通，水绿文一体和谐”的“塞上水城”。

高标准规划城市。围绕建设生态旅游文化城市，引入现代城市设计理念，科学地把现代文明、历史神韵、自然特色融入城市规划、设计和建设中，优化城市空间布局，完善新区控制性详规，做好城市水系规划、旧城改造规划、街景规划、地下人防工程建设规划等各专项规划的完善和衔接，进一步完善中宁、海原县城总体规划和控制性详规，打造亮点，彰显特色。严格规划管理，坚决查处各类违规建设行为，确保规划的严肃性。

高质量建设城市。举全市之力加快沙坡头机场建设，尽快打通中卫与外界的空中经济走廊，构筑现代化立体交通网，打造 “水、陆、空”三位一体的立体经济。深度开发利用黄河湿地公园、新区生态湖、腾格里湖等大型湿地湖泊，贯通沙坡头大道景观水道，沿生态湖规划建设明清仿古街等特色旅游商贸设施，改造提升老城区，开发建设新城区。完善各公园、广场、道路绿化景观，争取开工建设植物园等生态风景园区。实施环城东西路延伸、滨河路拓宽、水城路、环湖西路、府前路等主、次干道建设工程和新区供排水、供热、供电等公共服务设施项目，形成贯通东西、连接南北、便捷通畅的城市路网和配套完善的供排水、供热、供气管网。加快新区行政办公区和生态公园建设。实施“两街”（文昌街、长城街）、“两巷”（清真寺巷、东方红巷）改造工程。启动廉租房制度，适当增加经济适用房，抓好东方家园、官桥花园等15个旧城改造项目。维护住房公积金所有者合法权益，提高城镇居民居住水平。加快实施中宁县育才南路、供水厂续建、文化活动中心、南河子花园二期等38个重点项目和海原县城西环路、集中供热、污水处理、牌路山生态公园等项目。加快发展常乐、石空、兴仁等一批工矿型、商贸型、旅游型等各具特色的小城镇，构建区域发展平台，吸引农民向小城镇和非农产业转移。

高水平经营城市。强化资本运作意识，加大市场化力度，用好用活各类城市资源，广泛吸引外资和社会资金参与城市建设与经营。创新道路保洁、绿化管护等公用事业管理，加快供排水、公共交通、桥梁、道路、市政公用设施建设的市场化步伐，全方位、多途径推进城市资产优化配置与整合，提高综合效益和服务水平。

高效能管理城市。坚持“建管并举、重在管理”的方针，围绕提高市民素质、改善人居环境和提升城市品位，理顺城市管理体制，建立健全管理城市的长效机制。大力推进城市综合执法，深入开展“园林单位”创建、环境综合整治等活动，搞好环境卫生和绿化、亮化等公益基础设施建设，全面提高绿化、净化、美化、亮化水平，不断提升城市品位。

（四）多元发展第三产业，激发构建和谐中卫的发展活力

按照“发展大旅游、培育大市场、搞活大流通”的思路，整合资源，激活要素，发展壮大第三产业，切实增强经济发展活力。

发展大旅游。充分发挥旅游资源优势和区位优势，整合旅游资源，打造优势品牌，加快对外开放，构建旅游名市，使旅游成为我市靓丽的“名片”。强化旅游基础设施，加快建设沙坡头、寺口子、中宁双龙山、长山头天湖、海原九彩拱北等景区景点，大力发展农家乐等旅游项目，着力打造黄河漂流、沙漠探险、古刹建筑、生态观光、回乡风情、登山攀岩六大精品，拉长旅游链条，力争使沙坡头跨入国家5A级景区行列。依托丰富的人文资源，大力发展旅游文化产业，提高旅游与文化的融合度。注重横向联合，广泛开展区域旅游协作，加强与东部和中部旅游市场的对接，实现优势互补，继续办好 “中国宁夏（沙坡头）大漠·黄河国际旅游节”、宁夏中卫香山硒砂瓜节、“中宁枸杞节”等节事活动，增强吸引力和整体竞争力。全市旅游人数达到126万人次，实现旅游总收入3.5亿元，直接从业人员达到2200人。

培育大市场。加快市区和中宁、海原县城专业市场规划建设，加快建设中卫市场、中卫建材批发市场等各类市场和商贸园区。积极培育壮大房地产市场，新增商住面积40万平方米。继续实施“万村千乡”市场工程，进一步完善农村流通网络。严厉打击制售假冒伪劣商品的违法行为，维护良好的市场秩序。

搞活大流通。围绕建设宁夏乃至西部重要的商贸物流中心，依托沙坡头机场建设，整合周边空间资源，为沿黄城市带活跃的经济活动提供高效的人流、物流、资金流、信息流服务，实现中卫与外界的便捷对接。改造提升传统服务业，积极扶持发展物流配送、连锁经营、电子商务等现代服务业，繁荣活跃商贸流通。全社会消费品零售总额达到19亿元，增长11%。

（五）着力深化改革开放，增强构建和谐中卫的前进动力

坚持市场经济的改革方向，着力推进体制创新；坚持借力发展的开放方针，强力招商引资。以大改革促大开放，以大开放促大发展。

深化改革创新体制。坚持把改革放在更加突出的位置，奋力推进体制机制创新，增强经济发展活力。全面推进事业单位改革。继续深化企业产权制度改革，加快企业产权重组，实现股权结构多元化、治理结构现代化。实施农村综合改革试点，建立新型镇乡财政管理体制，逐步化解乡村债务。坚持依法治税，强化税收征管，推行社会综合治税，做到应收尽收。全面深化部门预算和国库集中收付改革，强化财政专项资金管理和企事业单位国有资产监督管理，优化财政支出结构。加强信用体系建设，营造良好的金融发展环境。加强银政、银企合作，充分发挥金融对地方经济发展的支持作用。

优化环境强力招商。牢固树立“环境创造财富，招商促进发展”的理念，进一步优化投资环境，全力招商引资。大力营造宽松的发展环境。全面兑现承诺的优惠政策，营造宽松和谐的政策环境；依法打击影响企业发展的不法行为，营造公平公正的法制环境；不断规范涉企行政行为，营造廉洁高效的政务环境；大力整顿规范市场经济秩序，营造诚实守信的市场环境，真正做到外来企业引得进、留得住、变得强。全面提高招商引资实效。创新招商方式，努力在产业招商、以商招商、资源招商上实现新突破。建立招商引资统筹机制。实行项目责任制、流失责任追究制，提高项目签约率和资金到位率，确保全年引资超过16亿元。

（六）全面繁荣社会事业，创优构建和谐中卫的人文环境

深入实施“科教兴市”战略。大力推进国家可持续发展实验区工程，围绕优势特色产业开展科技创新与推广活动。加快实施科技入户工程，积极推进中卫城区国家级和中宁县自治区级科技入户示范县建设。加大科普力度，不断提高群众科学文化素质。坚持教育优先发展，注重教育公平，提升教育整体水平。围绕“基础教育出品牌、职业教育创特色”目标，进一步优化配置教育资源，实施名师、名校、名校长工程，强化基础教育，提升高中教育，壮大职业教育，发展民办教育。建成中卫中学、海原职业教育中心，建设中卫职业教育中心，迁建中卫一小、中宁县中心小学。强化人才队伍建设，加大人力资源开发投入，注重对各级各类人才的培养、引进和使用，推进市场配置人才资源，吸纳更多的技术性人才落户中卫。

协调发展各项社会事业。加强城乡、社区文化体育设施建设，做好文化艺术中心、体育馆、海原地震博物馆建设工作。强化文化市场管理，积极发展文化体育事业和文化产业，不断提升群众素质。广泛开展全民健身活动，提高竞技体育水平，办好首届全国沙漠体育运动会。加强公共卫生、农村卫生和城市社区卫生工作，全面实施新型农村合作医疗，认真落实药品采购“三统一”，着力解决群众“看病难、看病贵”的问题。强化食品药品监管，完善公共卫生服务体系，提高疾病预防控制和突发公共卫生事件应急处置能力。做好第二次农业普查。进一步加强防震减灾、气象、物价、人防、档案、地方志等工作。

促进人与自然和谐。严格执行计划生育政策，稳定低生育水平，人口自然增长率控制在12.5‰以内。依法保护和合理利用国土资源。着力加强矿产资源开发的规划和管理，坚决制止滥采乱挖和严重浪费现象。进一步强化水资源保护和节约利用。认真执行环境影响评价和“三同时”制度，不断改善城市环境质量，提高可持续发展能力，努力建设资源节约型、环境友好型社会，实现人口、资源、环境协调发展，主要污染物排放量下降2%。

巩固社会和谐的思想道德基础。树立以“八荣八耻”为主要内容的社会主义荣辱观，倡导爱国、敬业、诚信、友善等道德规范，开展社会公德、职业道德、家庭美德教育，加强青少年思想道德建设，广泛开展和谐创建活动，在全社会形成知荣辱、讲正气、促和谐的良好风尚和男女平等、尊老爱幼、扶贫济困、礼让宽容的人际关系。弘扬科学精神，养成健康文明的生活方式。发扬艰苦奋斗精神，提倡勤俭节约。倡导和谐理念，培育和谐精神，形成符合传统美德和时代精神的道德规范和行为规范。把握正确舆论导向，加大外宣力度，提高中卫知名度。深入开展“五五”普法教育活动，切实增强全社会法制意识。巩固和壮大最广泛的爱国统一战线。加强与工会、共青团、妇联等人民团体的联系。进一步做好民族、宗教和侨务工作。强化国防教育，增强全民国防意识。巩固“双拥”成果，努力创建全国“双拥模范城”。

（七）高度关注民计民生，筑牢构建和谐中卫的民本基础

不断扩大城乡就业。营造全民创业的良好舆论氛围和社会环境，实施积极的就业政策，扶持百姓创家业，支持能人创企业，吸引中卫籍在外人士回乡创新业，促进创造活力竞相迸发、创业精英不断涌现、创业财富充分涌流。完善创业就业培训机制，大力开展各种类型的技术和职业技能培训，实施好“百万农民培训工程”和“农村劳动力转移培训阳光工程”，确保新增就业5000人、再就业400人，“零就业”家庭至少1人就业，城镇登记失业率控制在4.2%以内。

加快完善社保体系。多渠道筹集社会保障基金，加强基金监管，确保社会保险基金保值增值。逐步建立农村最低生活保障制度。完善城镇职工基本医疗保险，建立以大病统筹为主的城镇居民医疗保险，发展社会医疗救助。推进养老、失业、工伤保险制度建设。加强对困难群众的救助，完善城市低保、特困户救助、灾民救助等制度。发展以扶老、助残、救孤、济困为重点的社会福利。发展残疾人事业，保障残疾人合法权益。发展老龄事业，强化老龄服务，建设老年公寓。发展慈善事业，开展多种形式的捐助，增强全社会慈善意识。

切实维护公共安全。建立健全公共事件应急处理机制，构建预防化解社会矛盾的长效机制，切实加强信访工作，进一步畅通诉求渠道。高度重视安全生产，坚持“安全第一、预防为主、综合治理”的方针，全面落实安全生产责任制，深化安全生产专项整治，完善事故应急救援体系，最大限度预防和减少安全生产事故发生，确保人民群众生命财产安全。加强社会治安综合治理，巩固、深化、提升“平安中卫”创建活动，继续开展“打黑除恶”专项斗争，严厉打击各类违法犯罪活动，确保公共安全和社会稳定。

（八）切实加强行政能力，形成构建和谐中卫的有力保障

牢固树立服务、责任、诚信和法治理念，加快转变政府职能，着力推进管理创新，竭尽全力把中卫的事情办好，努力建设人民满意的政府。

增强科学施政的能力。牢记学习责任，确立终身学习理念，不断扩大视野，更新知识，进一步提高政府工作人员的政治素质、理论水平和执政能力。牢记创新责任，把与时俱进、开拓创新贯穿于政府工作全过程，科学判断形势，理性对待发展，深入基层、深入群众，注重用基层的经验指导实际工作，用个性的办法化解共性的难题，提升科学发展的水平和层次。牢记落实责任，切实把改革、发展、稳定的各项任务落到实处。

增强依法行政的能力。贯彻落实《行政许可法》和《全面推进依法行政实施纲要》，健全权责明确、行为规范、监督有效、保障有力的行政执法体制，确保行政决策和执法行为符合法定程序，促进和约束行政执法人员严格、公正、文明执法，不断提高政府依法行政水平。主动接受人大的法律监督、工作监督和政协的民主监督，自觉接受舆论监督、群众监督，认真办理人大代表议案、建议和政协提案，认真听取各民主党派、工商联、无党派人士和社会各界的意见建议，让政府的一切权力在阳光下运行。巩固扩大政务公开，提高政府工作的透明度和公信力。

增强为民执政的能力。时刻牢记党和人民的重托，牢记富民强市的重责，牢记跨越式发展的重任，扎扎实实干好本职工作。要敢于承担责任，在其位、谋其政、尽其责，干实事、求实效、促发展，上为组织分忧，下为百姓解难。要敢于吃苦耐劳，乐于奉献，带着感情、带着温暖、带着责任为群众服务，满腔热情地为群众办实事、解难题。要进一步完善公务员考核激励机制，促进政府全体工作人员精心谋事、潜心干事、坦荡处事、尽职办事，做到权为民用，情为民系，利为民谋。

增强从严治政的能力。坚持标本兼治、综合治理、惩防并举、注重预防的方针，推进教育、制度、监督并重的惩治和预防腐败体系建设。深入开展机关效能建设和治理商业贿赂工作，认真落实党风廉政建设责任制，坚持用制度管权、用制度管事、用制度管人、用制度管钱，强化对重大工程、政府采购和土地、矿产资源招投标等重点领域的管理和监察，坚决纠正部门和行业不正之风，切实从源头上预防和治理腐败。坚决纠正损害群众利益的不正之风，以清廉严明的形象取信于民。

各位代表！

成就载入史册，精彩献给未来。让我们高举邓小平理论和“三个代表”重要思想伟大旗帜，全面落实科学发展观，在自治区党委、政府和市委的领导下，紧紧依靠全市人民，以昂扬的斗志、饱满的热情、顽强的毅力、务实的作风，万众一心，奋发图强，锐意进取，扎实苦干，进一步唱响正气之歌、团结之歌、发展之歌、和谐之歌，为建设中卫美好的明天而努力奋斗！
